# 60th Annual Meeting Presidential Address

**DOI:** 10.1007/s43390-025-01261-2

**Published:** 2026-01-19

**Authors:** Laurel C. Blakemore

**Affiliations:** Scoliosis Research Society, Milwaukee, WI USA

Good morning, colleagues, friends, and members of the Scoliosis Research Society.

## Introduction

It’s been a profound honor and privilege to serve as SRS President. Each year we take this opportunity to reflect on where we are, celebrate the progress we’ve made, and share our vision for where we'll go next. So I’d like to speak with you about the state of the society and our accomplishments over the past year that we should all be proud of, as well as some of the challenges we face and where we’re going.

A wise predecessor said, “start with gratitude”- and I have that in abundance: for Ashtin and her team, for the board and committee chairs for their hard work, and for the PL who are generous with their support, friendship and wise advice. In a meeting this week, Rob Eastlack described the “cognitive might” of the SRS, and if you work with these leaders you will definitely feel it.

Kristen Jones and Meric Encercan led the creation of a program full of innovation for IMAST, and Jen Bauer, Martin Repko and the Program Committee, along with Noelle Larson and the Èducation committee have developed this year’s outstanding annual meeting content.

The friendships I’ve made here have been the most important of my career, and this deep collegiality is the secret sauce of the SRS. To my all time favorite SRS member, my husband Stephen Ondra, I’m grateful to you for sharing our wonderful family, your valuable wisdom, hours of healthcare policy education and your unfailing support. Thank you.

## State of the society

Through outstanding fiscal management by Ashtin and the EDI team, under the oversight of our Executive Committee, I am pleased to report that our Society has regained financial health. Our budget is balanced, and our stewardship of resources has been both thoughtful and strategic.

Meeting attendance has been strong, with our IMAST and Annual Meetings drawing increasing numbers again in 2025 despite turmoil and uncertainty in many regions, reflecting not only the scientific value of our meetings but also the community that binds us together. In an era where professional travel is scrutinized and resources are tight, this commitment speaks volumes about the vitality and value of SRS meetings.

Research remains the keystone of our society. Over the past five years, we have awarded a significant amount of research funding—more than 3 million dollars in 70 research grants, which have generated a quarter of a million dollars in additional funding and more than 130 presentations and publications. This impressive body of work continues to shape the field and inform clinical practice worldwide. We continue to lead and empower multicenter collaborations and provide the infrastructure for transformative studies.

As an example, the ASLS study which we have celebrated this week has produced a total of 25 manuscripts to date, 15 of which were supported by the SRS & sponsors, and much of the support was made possible by the generous donations of Dr. Lenke.

This is the kind of return on investment that changes patient care—and it is exactly why your contributions to the SRS- of time, treasure, and sweat—are so important.

We remain vigilant in protecting the Society’s long-term sustainability. As we work towards an endowment that helps us maintain financial stability, we continue to share a common mission with our industry partners and work together to improve outcomes for our patients.

## Partnerships and collaboration—building the ecosystem

Much of our ongoing work this year has focused on collaboration and partnerships. We are in a changing world and our success depends on understanding our value: to our members, and to all of the stakeholders in our complex ecosystem. We’re unique in spine care because we are a truly global society, and our strength is amplified through partnership with other societies and in other regions.

This year we’ve collaborated on educational efforts with partners like Eurospine, CNS, and Joint Section, building bridges that allow our programs to reach more people, expand our influence and create opportunities for deeper collaboration. As part of a leadership retreat this spring we initiated a process for partnership assessment, so that we can make informed and strategic decisions about when and how to invest resources in these efforts and how they add value for our members. We are exploring a stronger partnership with our colleagues at Eurospine that we think will amplify our educational reach in complex spine.

In advocacy, our leaders have done tremendous work to help us clarify our role and our communications. Our Position Statement Task Force led by Michelle Welborn and Communication Council Chair Ron El Hawary has developed a comprehensive framework to provide direction for our committees, producing statements and for our publications on policy and innovation, as well as define the process and resources needed to create this work. This gives us robust guidelines for what we want to say, why we should say it and what resources we need to create it.

This year we also continued to engage actively with all stakeholders surrounding European Medical Device Regulation, building on the progress made by Pat Cahill, Noelle Larson and others whose work with the FDA through the Pediatric Device Task Force has made real gains for device regulation in the US. Marinus de Kleuver and others have proactively led roundtable discussions and educational sessions at IMAST and the annual meeting. Our role has been as a connector, engaging all stakeholders, and potentially engaging and educating regulatory bodies to ensure that our patients have timely access to safe, effective treatments and innovations.

Together, all of these efforts create what is our ecosystem—an interconnected network of individuals, institutions, and partners united by the shared goal of improving outcomes for patients with spinal deformity. As a global society we span cultures and countries across the globe, tied together by our shared mission of improving the lives of our patients.

## Where are we going? (No resting on our laurels)

As proud as we are of our past and present, we have many challenges to overcome. The future of the SRS depends on inclusion, leadership, and vision. Peter Newton’s Harrington lecture highlighted the importance of objectivity and vision as we look forward to where the SRS is going and what we’re doing.

### LEAD SRS

The SRS has high standards for ourselves, our research and our educational offerings. For SRS to thrive, we must continue to attract the best and most passionate people in orthopedics, neurosurgery, research and comprehensive care.

We want *all* of them to see SRS as their professional home- as the best source for the tools they need to provide outstanding care for patients and continually make that care better. Diverse teams produce stronger science, more innovative solutions, and more compassionate care. We’ll meet our mission by welcoming and supporting outstanding members from every discipline, region, and background, and the Leadership Education and Development (LEAD) program developed by Serena Hu and Todd Albert is one of the best ways to help foster our future: a group of leaders that ensures the SRS will continue to thrive. Members also support up and coming leaders through multiple grants including the Travelling Fellowship, The Yazici fund, and the Lori Karol Women in Spine Fund.

Last year Marinus de Kleuver discussed Physician Wellness and how important our own health is to our ability to provide outstanding care. Over the past year Marinus, Ryan Fitzgerald and John Vorhies have designed a new SRS Peer Support program that we’ll be introducing later this year. The SRS will pair members who have experienced an adverse patient event with volunteer peer supporter who will have completed comprehensive training that allows them to provide a safe and supportive space for physicians to process their experiences, share their burdens, and find a path forward.

### Lead in education

Education is core to who we are as a society. We continually assess our educational programs for value, relevance and sustainability to respond to our members and attendees. Our precourse this year was a great example of how we help frame complex spine care in an era of rapidly advancing technology and global shifts in health care. We lead efforts to offer spine deformity training across the globe. I think the SRS will also help lead the conversation on spine fellowship training as we navigate the shifting landscape in complex spine care in many regions, and I look forward to being a part of that work.

We are also expanding our accessibility in Asia Pacific with a program that’s “by the region, for the region”, and will feature faculty and participation from 14 Asian spine societies. Led by regional SRS leaders, this meeting will feature the best papers from the participating societies and serve as a pilot that can inform future programming in other regions of the world.

### Closing

I began by telling you what an honor and privilege it has been to serve as SRS president this year. This community of dedicated, brilliant individuals has been my second family. But leadership isn’t about one year, it’s part of a continuum that goes from the beginning and purpose of a society and extends into the future. Each year is a chance to carry the baton for part of that journey. It has been the greatest honor of my professional life to carry that baton and serve the society. Together, we have balanced budgets, funded research, built partnerships, and strengthened our voice in advocacy. But our greatest achievements lie ahead—and there is no doubt that the leaders among you today will take the SRS across all bridges and beyond. Please join us in Sydney next year for the next chapter!

Thank you.

Laurel C. Blakemore, MD

55th President, Scoliosis Research Society

60TH Annual Meeting Presidential Address, Charlotte, North Carolina

September 19, 2025.

